# Monthly Summary

**Published:** 1892-12

**Authors:** 


### Monthly Summary.

GOLD WASTE OF VALUE.—When the American Waltham Watch Company moved to new quarters in May last, they left behind a snug fortune in the old buildings in Bond Street, this city, in the shape of minute particles of gold among the old rubbish and in the cracks of the pine flooring. The precious metal has been reclaimed, however, by the Irvington Smelting and Refining Works, at Irvington, N. J., in the form of bars of yellow gold. The total value of the gold reclaimed is between $65,000 and $67,000. The gold recovered must have accumulated since 1879, when the factory was occupied by the firm. An average of 350 gold watch-cases were turned out every day, each case weighing from twenty to fifty pennyweights. The gold was valued at eighty cents a pennyweight, and during a year it was estimated that more than $500,000 worth of gold was used in the manufacture of watch-cases.

The water in which the workmen washed their hands, the mats on which they walked, and the towels with which they dried their hands and faces were carefully preserved, and at the end of every month were strained or burned and the residue afterward smelted and refined. About $1,000 a month was saved in this manner. Every summer parts of the flooring were taken up and smelted, and sometimes as much as $7,000 was realized in this way.

To obtain the gold concealed in the cracks and crevices in the old building, wagons were especially made to cart the old material from the factory to the smelting works, and every stick of wood in their premises was taken away. The planks of the floors were sawed into small pieces and then burned. The ashes were subjected to a chemical wash and the gold extracted.—*Scientific American.*

---

ARROGANT CRITICS.—Every thinking man is more or less a critic. But there are critics and critics. When a new idea, or an old one renovated, is presented for discussion, some critics will examine it with but one object—the unselfish desire to get at the truth. Others will measure it by their one and only standard of mathematical certainty—its exact correspondence with their own preconceptions. Dentistry, like other occupations, is not exempt from the professional Thersites, who love to bring the modest efforts of their fellow-workers into ridicule; who set themselves up, not only as connoisseurs, but as professional scolds, and who think "when they ope their mouths, no dog should bark."

One cannot be too severe upon the advertising quack and impostor, but it is discouraging to men who mean well, but who do not assume infallibility, so to find their humble efforts sneered at by some arrogant critic, who is never happy but when burning the incense of admiration before his own productions.

As a rule, the truly great men in any sphere are not those who find it necessary to depreciate thought and labor they have not themselves performed. The truly great are those who welcome every honest search for the truth, and whose criticism is crowned by their charity. Many a worthy young man is deterred from literary and scientific effort in associations by the stupendous arrogance of some self-elected "Great I am," whose over-bearing conceit blinds him to the fact that another man may possibly be right, and he altogether wrong. The profession is, however, full of generous critics and noble men, who make no pretence themselves to omniscience, and who do not expect it in others. We have pleasant recollections of the charity such men extend to their confreres, in personal associations with the Odontological Society of New York, and no doubt the success of that illustrious body is due not only to the zeal and ability of its members, but to the fact that in criticism they never forget they are gentlemen.—*Dominion Dental Journal*..

---

ARTIFICIAL INDIA RUBBER.—Dr. W. A. Tilden discovered some months ago that isoprene, which can be prepared from turpentine, under certain circumstances changes into what appears to be genuine India rubber. Bouchardat had also found that the same change could be brought about by heat. The material so produced resembles pure Para rubber in every way, and, whether it is genuine rubber or not, it may be equally good for all practical purposes. It is said to be capable of vulcanization.—*Scientific American*.

---

ANOTHER CASE OF INSANITY CAUSED BY NITROUS OXIDE GAS.—Miss Lizzie Lots, the beautiful daughter of Mr. Otto Lots, a prominent citizen of Covington, Ky., is now at College Hill Sanitarium receiving treatment for her mind. The family claim that Miss Lots became insane through taking nitrous oxide gas for the extraction of two teeth while visiting at Indianapolis

Her sister noticed her candition of mind, and immediately sent her home. She seemed to be all right for a few days, but occasionally claimed that she had gas in her head. She would tell her father that he was to be the next President of the United States, and that he would reappoint Mr. Hardeman as postmaster of Covington. Again, she would talk on all sorts of financial schemes of great magnitude. Her condition finally became so alarming that her folks were compelled to remove her to the sanitarium for treatment. She now speaks of the sudden spells she had at home. Her father is confident that her malady was caused by the taking of gas and pulling of her teeth. He declares that the girl was never sick in her life before, and that her mind became impaired immediately after she had her teeth extracted.

Mr. Lots is borne out in his statements by his other daughter, who lives at Indianapolis. She is a favorite child, and her venerable father is almost prostrated over her condition. He has given her all the medical attention that he can, but grave fears are entertained as to her ultimate recovery. She is well known among the people of Covington, and had a successful business. Her father is enraged over the treatment she received at the hands of the dentist in Indianapolis, and he has employed Senator Goeble, of Covington, to bring an action for damages against him.

Miss Lots is a striking beauty and highly accomplished in music. She is about twenty-two years of age, and a brunette of purest type. She is very popular among all her acquaintances, who will be surprised to learn of her distressing malady. Her parents reside at 1003 Greenup street, Covington.

---

DEATH IN A DENTIST'S CHAIR.—Mrs. Philip Sebest, a comely married woman, met death in a dentist's chair in Elmira, N. Y., recently. The cause of her death is not fully accounted for by the physicians, as the autopsy showed her heart to be in a normal condition, and a combination of causes was the verdict of the doctors. Shortly before five o'clock Mrs. Sebest went to the office of Dr. G. H. Preston, on West Water street, to have some teeth extracted. He administered nitrous oxide gas, and placed his nippers upon the tooth to be extracted. Mrs. Sebest said to the dentist that she felt the pain, and wanted the dentist to administer more gas, which he refused to do. When the tooth was drawn she uttered a scream, and spat some blood in the cuspidor, gasped, and fell back in the chair, dead. Drs. C. W. M. and M. M. Brown were summoned, and also Coroner Westlake who made an examination. It is claimed that Dr. Preston had before, on several occasions, administered gas to Mrs. Sebest without injury, and it is thought that the excessive heat, pain and excitement brought on a stroke of apoplexy. Mrs. Sebest's sister was with her at the time the operation took place, and certified to the fact of the dentist's refusal to administer gas a second time.—*Items of Interest.*

---

DEATH FROM SYNCOPE.—On the 9th of August last, Dr. E. A. Guilbert, a prominent physician of Dubuque, Iowa, gave what is known as the A. C. E. mixture, to Mrs. J. C. Fitzpatrick, of same city, aged about thirty years, in good health, for the painless extraction of teeth, in the office of Dr. J. V. Conzett, a prominent young dentist in Dubuque, who extracted the teeth. The lady is thought to have been under the influence of the anæsthetic about twenty minutes, and yet was capable of resisting the efforts of the dentist at least part of that time, and seemed to have partially recovered from the effects of the anæsthetic, when all of a sudden she ceased breathing.

We further learned from both the dentist and physician, that there was positively no purpleness nor deathly pallor till after the functions of life had ceased, which is abundant evidence that the patient was not asphyxiated by the anæsthetic.

Again, it was quite a little time after the anæsthetic had been administered, before the heart and lungs refused to perform their functions, which precludes the possibility of the death from what is commonly called a shock from the chemical action of the drug. Therefore, if neither asphyxia, nor paralysis of nerve center by the anæsthetic, were produced, then the anæsthetic could not have killed the patient. Hence, the patient must have died from syncopy, with partial narcosis. As syncope is always produced by the mind, or an acquiescent condition of the mind, the probability is that the strange sensations of the anæsthetic as felt as the patient was recovering consciousness, caused fright and syncope, which with partial narcosis, is a very unsavory condition, and should always be obviated by the administrator describing the manifestation of the anæsthetic. If this be neglected before administering, the patient having heard of deaths from anæsthetics, or so-called anæsthetics, would very naturally think that the pricking sensation and numbness, or strange, cold, numb feeling creeping all over, were but the chills of death, and be terribly frightened and faint.

The only censure in the above case would be for not having the corsets and skirts properly loosened, and the necessary agencies at hand to resuscitate the patient, and perhaps not having properly described the manifestations of the anæsthetic when about to administer. Yet it is due the physician and dentist that when they discovered the perilious condition of their patient, that they used every available means of resuscitation, and yet without success.—*D. & S. Microcosm.*

---

TREATING AN EXPOSED, OR PARTLY EXPOSED, PULP.—First saturate the cavity with "heaven's" cordial, and a little of the following mixed with it; one part oil cloves, three parts carbolic acid made into a soft paste with tannin. If you have not heaven's cordial, use in its place chloroform. Then clean away all debris from the cavity, as well as you can, without disturbing the pulp. Be careful not even to lift the thin, leathery layers of softened dentine from over or near it; for the less the pulp is disturbed the surer the treatment. Now, cut down weak walls, and be as thorough in preparing the cavity for filling as possible, without impinging on the pulp. Rub a little of the obtundent in the cavity (but not enough to saturate), and over the flow a coating of chloropercha. Blow on warm air to evaporate the chloroform, and then fill the cavity with oxyphosphate, put in rather thin, so as to avoid all pressure, and also so that it will stick to the walls of the cavity.

In from four to six weeks the pulp will be incased in a beautiful tanned covering. This should not be disturbed. The best way is to have everything intact, except to remove a little of the oxyphosphate, so as to give room and undercut for a permanent covering of alloy of gold.

Before this, however, and during the healing of the pulp, there may be an accumulation of pus. If so, remove the temporary filling, thoroughly syringe with hot water, and after flowing over a little peroxide of hydrogen, then repeat the first dressing, though of course a favorable result is not so sure.—*Items of Interest.*

---

SUDDEN DEATH.—Mrs. Kate Ledbetter, daughter of Peter McCue, who lives near Oakford, died in Dr. Solenberger's dental office Wednesday evening, while under the influence of an anæsthetic, which, at her request, had been administered by Dr. Whitley, preparatory to having some teeth pulled. Coroner McAtee held an inquest, at which it appeared in evidence that the usual precautions had been taken, before and during the administration of the anæsthetic, which Dr. Whitley stated was hydrobromic ethyl, used for short operations. The coroner's jury was composed of Z. A. Thompson, foreman; A. E. Estill, E. H. Bigelow, Mat. Hainsfurther, E. R. Oeltjen, H. C. Levering. They returned the following verdict.

"We, the undersigned, jurors, sworn to inquire of the death of Mrs. Kate Ledbetter, on oath do find that she came to her death by paralysis of the heart. We further exonerate all parties from blame."

The body was taken to the residence of J. D. Roberts, where it was prepared for burial by undertaker Conant, and taken to Oakford on Thursday morning's train, the funeral occurring yesterday.

The deceased was the wife of Frank Ledbetter, a Wyoming ranchman, and was here on a visit. Her husband was coming through to Chicago with a shipment of stock and was to stop here on his way home. Mrs. Ledbetter was about thirty years of age.—*Democrat*, Petersburg, Ill.

[The above verdict is a sad comment upon the intelligence of the coroner's jury. Did ever any animal or man die without the heart being paralyzed? The object of a coroner's jury is to ascertain the cause of the death, if possible, and to designate the cause in their verdict. The lady died, but what caused the paralysis of the heart or death? Was it the drug administered, or the manner of making and administering the anæsthetic that caused the death; or did she die from syncope? Why not give an intelligent account of the administration and effects of the agency, and of what was done to save the life of the patient? These grave offences should not be covered up. Death under such circumstances should not be winked at or the cause withheld from the public.—*D. & S. Microcosm.*]

---

PROFESSIONAL DIGNITY.—The relation of dentist to patient is peculiar, sometimes complex, occasionally embarrassing. Its tendency to the social, the sympathetic, and even to the pitiful, increases its delicacy, and whispers caution. Yet it is not difficult to an operator of sound mind and heart, and good common sense. If he is a true man, awkwardness will soon graduate to refinement, blunders to correct deportment, and embarrassment to ease. Experiment and a delicate discernment will tell him where to walk softly, when to use diplomacy, and how to produce trustfulness. But it is well for us all to keep in mind the difference between dignified familiarity and undignified intimacy. We must have an intuitive perception and an insinuating delicacy not developed in a coarse, vulgar nature. But where there is refinement there will be discretion that is pleasing, a delicacy of language and deportment that is winning, and a flow of wit, or of intelligence, or of society, that brings friendship, begets confidence and mitigates suffering.— ED. *Items of Interest.*

